# Supplementary material for: Isolation and Characterization of the Acadevirus Members BigMira and MidiMira Infecting a Highly Pathogenic Proteus mirabilis Strain
Source: Microorganisms. 2023 Aug 23;11(9):2141. doi: 10.3390/microorganisms11092141 (PMC10537623; doi:10.3390/microorganisms11092141)
Supplement: Supplementary file 1 [file microorganisms-11-02141-s001.zip › microorganisms-2478866-supplementary.pdf]

Isolation and characterization of the *Acadevirus* members BigMira and MidiMira, infecting a highly pathogenic *Proteus mirabilis* strain

Jéssica Duarte da Silva[1], Lene Bens[2], Adriele Jéssica do Carmo[1], Rob Lavigne[2], José Júnior Ferreira Soares[1], Luís Daniel Rodrigues Melo[3], Marta Vallino[4], Roberto Sousa Dias[5], Zuzanna Drulis-Kawa[6], Sérgio Oliveira de Paula[1,5], Jeroen Wagemans[2*].

[1] Laboratory of Molecular Immunovirology, Department of Microbiology, Federal University of Viçosa, Viçosa, Brazil.

[2] Laboratory of Gene Technology, Department of Biosystems (Division of Animal and Human Health Engineering), KU Leuven, Leuven, Belgium.

[3] Centre of Biological Engineering, University of Minho, Braga, Portugal

[4] Institute for Sustainable Plant Protection, National Research Council of Italy, Torino, Italy.

[5] Department of General Biology, Federal University of Viçosa, Viçosa, Brazil.

[6] Department of Pathogen Biology and Immunology, University of Wroclaw, Wroclaw, Poland.

* Correspondence: jeroen.wagemans@kuleuven.be

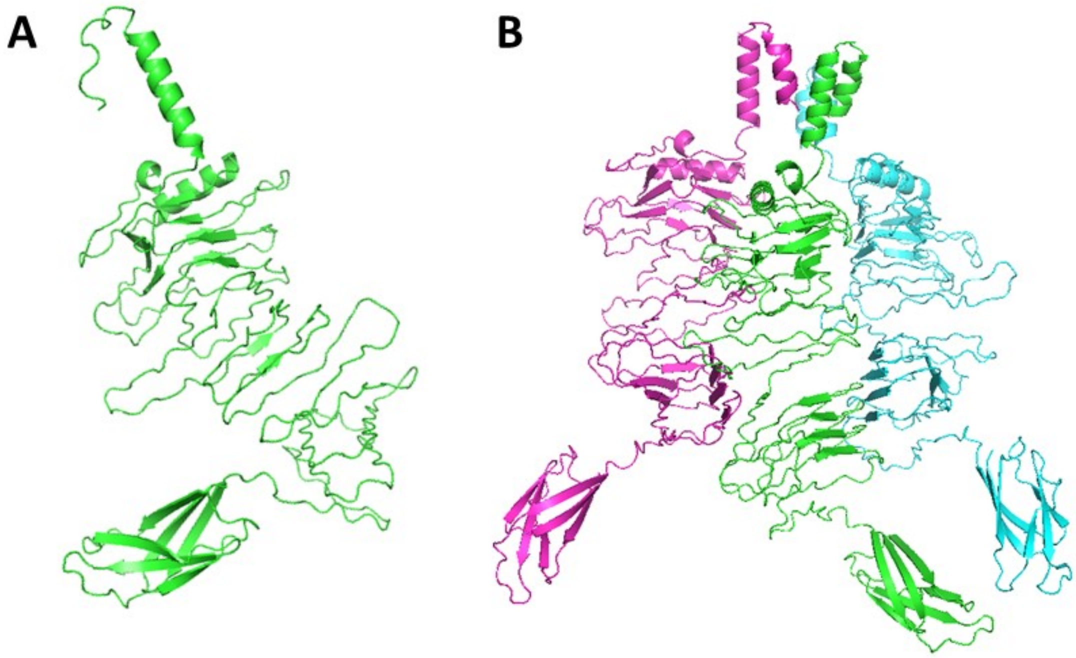

Figure S1: 3D prediction of Gp 52, a hypothetical protein. A. Monomer folding prediction. B. Trimer prediction.

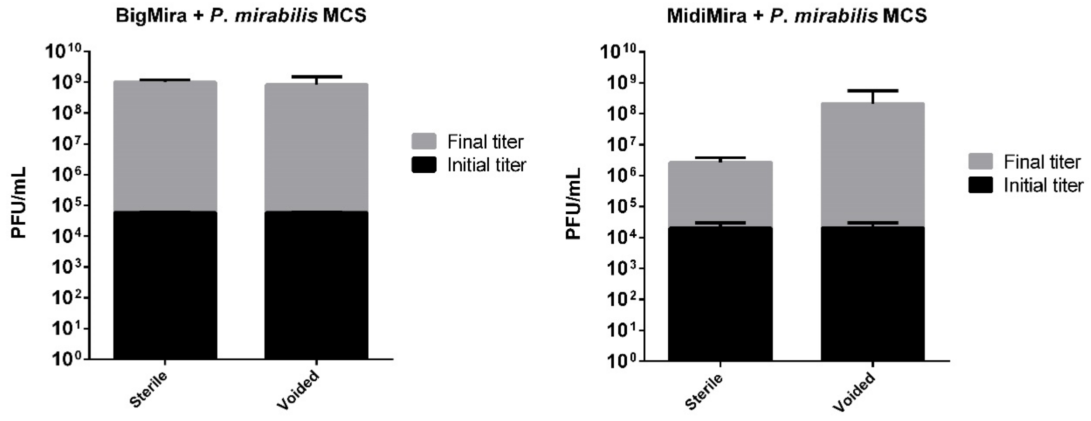

Figure S2: Phages BigMira and MidiMira propagation rate in sterile and voided urine after 24 hours of incubation.
